# Menthol response and adaptation in nociceptive-like and nonnociceptive-like neurons: role of protein kinases

**DOI:** 10.1186/1744-8069-6-47

**Published:** 2010-08-20

**Authors:** Ignacio Sarria, Jianguo Gu

**Affiliations:** 1 Department of Anesthesiology, University of Cincinnati College of Medicine, PO Box 670531, 231 Albert Sabin Way, Cincinnati, OH 45267-0531, USA; 2 Graduate Program in Neuroscience, University of Cincinnati College of Medicine, PO Box 670531, 231 Albert Sabin Way, Cincinnati, OH 45267-0531, USA

## Abstract

Menthol-sensitive/capsaicin-insensitive neurons (MS/CI) and menthol-sensitive/capsaicin-sensitive neurons (MS/CS) are thought to represent two functionally distinct populations of cold-sensing neurons that use TRPM8 receptors to convey innocuous and noxious cold information respectively. However, TRPM8-mediated responses have not been well characterized in these two neuron populations. Using rat dorsal root ganglion neurons, here we show that MS/CI neurons had larger menthol responses with greater adaptation. In contrast, MS/CS neurons had smaller menthol responses with less adaptation. All menthol-sensitive neurons showed significant reduction of menthol responses following the treatment of cells with the protein kinase C (PKC) activator PDBu (Phorbol 12,13-dibutyrate). PDBu-induced reduction of menthol responses was completely abolished in the presence of PKC inhibitors BIM (bisindolylmaleimide) or staurosporine. When menthol responses were examined in the presence of protein kinase inhibitors, it was found that the adaptation was significantly attenuated by either BIM or staurosporine and also by the Ca^2+^/calmodulin-dependent protein kinase **(**CamKII) inhibitor KN62 (N,O-bis(5-isoquinolinesulfonyl)-N-methyl-L-tyrosyl]-4-phenylpiperazine) in MS/CI neurons. In contrast, in MS/CS neurons menthol response was not affected significantly by BIM, staurosporine or KN62. In both MS/CI and MS/CS neurons, the menthol responses were not affected by PKA activators forskolin and 8-Br-cAMP (8-Bromoadenosine-3', 5'-cyclic monophosphate) or by protein kinase A (PKA) inhibitor Rp-cAMPs (Rp-Adenosine-3',5'-cyclic monophosphorothioate). Taken together, these results suggest that TRPM8-mediated responses are significantly different between non-nociceptive-like and nociceptive-like neurons.

## Background

Transient receptor potential M8 (TRPM8) receptor, first cloned by MacKemy and colleagues [1] as well as Peier and colleagues [2] from primary afferent neurons of rats and mice, is a principal sensor for cold temperature and belongs to the transient receptor potential (TRP) protein family. Like most of other members in TRP family, TRPM8 is a membrane ion channel that can allow positively charged ions (Na^+^, Ca^2+^, K^+^) to flow through cell membranes when the channel opens. The TRPM8 channel opens when temperature drops below 26 ± 2°C, resulting in depolarizing membrane currents [1-3]. Membrane currents flowing through TRPM8 channels increase with decreasing temperature and reach maximum response near 10°C. TRPM8 senses temperature changes in the range of both innocuous cold (28-15°C) and noxious cold (<15°C) [1-3]. Activation of TRPM8 can result in a large increase of intracellular Ca^2+ ^levels due to the high Ca^2+ ^permeability of this channel [1,2,4,5]. TRPM8 can also be activated by menthol, an active ingredient of peppermint that produces a cooling sensation [1,2,6,7].

TRPM8 receptors are expressed on 10-15% of the total trigeminal ganglion (TG) neuron population and 5-10% of dorsal root ganglion (DRG) neuron population [1,2,7,8]. Consistently, the percentage of menthol-sensitive cells in acutely dissociated rat DRG neurons is similar to that of TRPM8-expressing DRG neurons [9,10]. Many TRPM8-expression neurons are found to lack nociceptive markers, suggesting that they are non-nociceptive cold sensing neurons [2]. However, studies have provided anatomical evidence showing TRPM8 immunoreactivity on some TRPV1 (Transient receptor potential V1)-expressing afferent neurons [7,8]. TRPV1-expressing neurons are believed to be nociceptive afferent neurons that transmit noxious signals to produce burning pain sensations [11-13]. Using calcium imaging and patch-clamp recording techniques, Xing and colleagues [9] have found that a subpopulation of menthol-sensitive neurons is also sensitive to capsaicin, a noxious stimulant that acts on TRPV1 receptors. Consistent with these observations, co-expression of TRPM8 and TRPV1 have been directly visualized in mice engineered to express enhanced green fluorescent protein (EGFP) driven by a TRPM8 promoter [14,15]. Thus, menthol-sensitive neurons appear to consist of both non-nociceptive and nociceptive sensory neurons and may play roles in sensing innocuous and noxious cold respectively under physiological conditions [10].

TRPM8 can be regulated through second messenger systems [16-18]. A role for the PLC/PIP2 (Phospholipase C/phosphatidylinositol (4,5) bisphosphate) second messenger pathway in regulating TRPM8 functions has been well established [16,17,19]. It has been suggested that Ca^2+ ^influx through TRPM8 channels activates a Ca^2+^-sensitive phospholipase C and the subsequent depletion of PIP2 results in desensitization of TRPM8 channels [16,17,19]. Desensitization of TRPM8 channels could also be induced by inflammatory mediators that activate PLC to deplete PIP2 [20]. In comparison with the PLC/PIP2 pathway, the roles of protein kinase pathways in regulating TRPM8 functions remain unclear. Premkumar and colleagues [18] showed in DRG neurons that PKC activators and bradykinin significantly reduced menthol responses. Using HEK293 cells expressing TRPM8, Abe and colleagues [21] also showed that PKC activators reduced menthol responses. Other second message pathways such as PKA have also been suggested to play roles in regulating TRPM8 functions [22,23]. These previous studies on the regulation of TRPM8 functions were performed either using heterologous expression system or functionally unidentified sensory neurons. Therefore, it is unclear if the reduction of TRPM8 functions occurs in a similar manner across functionally distinct populations of neurons. In addition, previous studies did not test whether TRPM8-mediated responses were affected by different protein kinase inhibitors, a result that is essential for establishing the roles of protein kinases in modulating TRPM8 functions. In the present study, we addressed some of these issues by examining menthol-responsiveness and adaptation in menthol-sensitive/capsaicin-insensitive and menthol-sensitive/capsaicin-sensitive neurons.

## Methods

Adult Sprague Dawley rats (100-250 g, both genders) were used in all experiments. Animal care and use conformed to National Institutes of Health guidelines for care and use of experimental animals. Experimental protocols were approved by the University of Cincinnati Institutional Animal Care and Use Committee. DRG neuron cultures were prepared as described previously [4]. In brief, rats were deeply anesthetized with isoflurane (Henry Schein, NY.) and sacrificed by decapitation. DRGs were rapidly dissected out bilaterally in Leibovitz L-15 media (Fisher, GA) and incubated for 1 hour at 37°C in minimum essential medium for suspension culture (S-MEM) (Invitrogen, Grand Island, NY) with 2% collagenase and 10% dispase and then triturated to dissociate neurons. The dissociated DRG neurons were then plated on glass coverslips pre-coated with poly-D-lysine (PDL, 12.5 μg/ml in distilled H_2_O) and laminin (20 μg/ml in Hank's Buffered Salt Solution HBSS, BD bio-science), and maintained in MEM (Invitrogen) culture medium that also contained nerve growth factor (2.5 S NGF; 10 ng/ml; Roche Molecular Biochemicals, Indianapolis, IN), 5% heat-inactivated horse serum (JRH Biosciences, Lenexa, KS), uridine/5-fluoro-2'-deoxyuridine (10 μM), 8 mg/ml glucose, and 1% vitamin solution (Invitrogen). The cultures were maintained in an incubator at 37°C with a humidified atmosphere of 95% air and 5% CO_2_. Unless otherwise indicated, cells were used within 72 hours after plating.

For calcium imaging experiments, the calcium indicator Fluo-3 (Invitrogen) was loaded into DRG neurons on coverslips by incubation of cells with 5 μM Fluo-3-AM in normal bath solution at 37°C for 1 hour. Fluo-3-AM stock solution was made with 20% pluronic acid (Molecular Probes) in dimethyl sulfoxide (DMSO) and the stock solution was diluted 1:200 with bath solution for final use. Normal bath solution contained (in mM) 150 NaCl, 5 KCl, 2 MgCl_2_, 2 CaCl_2_, 10 glucose, 10 HEPES, pH 7.3 adjusted with NaOH, and osmolarity 320 mOsm adjusted with sucrose. After dye loading, a coverslip was mounted on a 0.5-ml perfusion chamber and the chamber was then placed on the stage of an inverted Olympus IX70 microscope (Lake Success, NY). Cells on the coverslip were continuously perfused with normal bath solution flowing at 1 ml/min. Fluo-3 was excited at 450 nm with a mercury lamp and fluorescence emission was collected at 550 nm, and the wave lengths of excitation and emission were achieved by a fluorescence filter set. Fluo-3 fluorescence in the cells was detected with a peltier-cooled charge-coupled device (CCD) camera (PentaMAX-III System, Roper Scientific, Trenton, NJ) under a 10× objective. Images were acquired at one frame per second, 200 ms exposure time per frame, using the MetaFluor Imaging System software (Molecular Devices, Downingtown, PA). Neurons were tested for their sensitivity to menthol (100 μM), AIT (allyl isothiocyanate, 100 μM), or capsaicin (0.5 μM) by applying these compounds for 10 seconds. Adaptation of menthol responses was examined by a prolonged application of menthol (100 μM) for 5 min. Effects of protein kinases on menthol responses and adaptation were tested with 1 μM PDBu (phorbol 12,13-dibutyrate), a protein kinase C (PKC) activator, 100 μM 8-Br-cAMP (8-bromoadenosine-3', 5'-cyclic monophosphate) and 10 μM forskolin, two protein kinase A (PKA) activators, 0.5 μM staurosporine, a broad spectrum protein kinase inhibitor, 1 μM BIM (bisindolylmaleimide), a specific PKC inhibitor, 25 μM RP-cAMPs (Rp-adenosine-3',5'-cyclic monophosphorothioate), a specific PKA inhibitor, and 25 μM KN-62 (1-[N,O-bis(5-isoquinolinesulfonyl)-N-methyl-L-tyrosyl]-4-phenylpiperazine, Tocris), a specific Ca^2+^/calmodulin-dependent protein kinase (CaMKII) inhibitor. Unless otherwise indicated, chemicals and compounds were purchased from Sigma (St. Louis, MO). Testing solutions were rapidly applied to neurons through a glass tube (~500 μm ID) positioned 1.0 mm away from cells. Unless otherwise indicated, testing compounds were applied at an interval of 10 min and capsaicin was always tested last. All experiments where pharmacological agents were applied were done in separate dishes. All experiments were carried out at room temperature of ~24°C.

For most experiments, relative fluorescence intensity (ΔF/F_0_) was used to represent menthol responses and neurons with ΔF/F_0 _values of ≥0.2 (i.e., equal or above 20% baseline fluorescence intensity) were considered as responsive cells [9]. Percentages of maximal ΔF/F_0 _values were used as changes in menthol responses. In some experiments, intracellular Ca^2+ ^concentrations ([Ca^2+^]) in cells were calibrated from the measured fluorescence signals. The following equation was use for the calibration of intracellular Ca^2+ ^concentrations [24]:

[Ca^2+^] = Kd[(F-Fmin)/(Fmax-F)] where [Ca^2+^] is the concentration (nM) of intracellular Ca^2+^, Kd is the dissociation constant of the dye, F is the fluorescence intensity, Fmin is the intensity at zero [Ca^2+^] and Fmax is the intensity at saturated [Ca^2+^]. Procedures for obtaining Fmax and Frnin caused damage to cells and were therefore carried out at the end of the experiments. Fmax was obtained first by adding the ionophore ionomycin (10 μM), making the cell membrane permeable to Ca^2+ ^and allowing the extracellular and intracellular Ca^2+ ^to equilibrate. Following this, Fmin was obtained by adding EGTA [ethylene glycol bis(~-aminoethyl ether)-N,N,N',N'-tetraacetic acid; 20 mM] to chelate all Ca^2+ ^inside and outside the cells. Then MnCl_2 _(30 mM) was added to quench the residual fluorescent signals due to autofluorescence [25]. Kd value of 404 nM was used according to a previous study [26]. Changes in intracellular Ca^2+ ^concentrations are calculated by Δ[Ca^2+^]/[Ca^2+^]_0, _where [Ca^2+^]_0 _is basal intracellular Ca^2+ ^level and Δ[Ca^2+^] is the difference between intracellular Ca^2+ ^concentrations at a giving time point and [Ca^2+^]_0_. Unless otherwise indicated, data were presented as Mean ± SEM. Analysis of Variance (ANOVA) was applied for statistic analysis of unpaired data sets of multiple groups followed by Student-Newman-Keuls Post Hoc Test. Student's t-test was applied for paired data sets. Statistical significance was considered at the level of the p < 0.05.

## Results

### Menthol responses and adaptation

**Figure 1:**
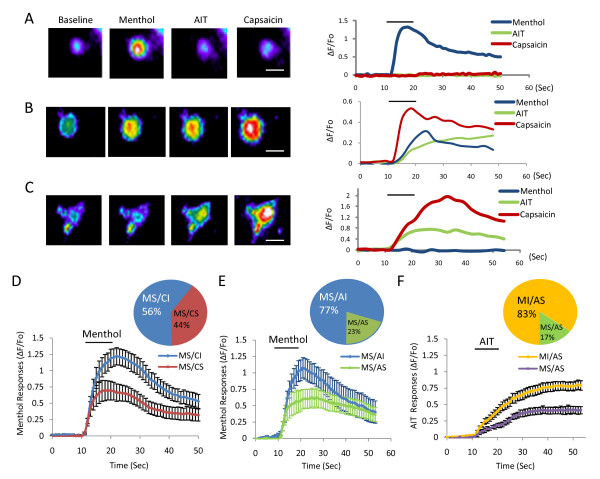
**Responses to menthol, capsaicin and AIT in dorsal root ganglion neurons of rats**. ***A, B, C*) **Images show Fluo-3 fluorescence intensity (ΔF/F_0_) before (baseline) and following the sequential applications of menthol (100 μM, 10 s), AIT (100 μM, 10 s), and capsaicin (0.5 μM, 10 s) with a time interval of 10 min between each application. Traces alongside with each set of images are respective time-course of fluorescence intensity (ΔF/F_0_) changes. The cell in **A **was a menthol-sensitive/AIT-insensitive/capsaicin-insensitive neuron and in **B **was a menthol-sensitive/AIT-sensitive/capsaicin-sensitive neuron. The cell in **C **was a menthol-insensitive/AIT-sensitive/capsaicin-sensitive neuron. ***D, E) ***Pie graphs in each panel show percentage distribution in total menthol-sensitive neurons of menthol-sensitive/capsaicin-insensitive neurons (MS/CI, n = 40) and menthol-sensitive/capsaicin-sensitive (MS/CS, n = 31) neurons **(D) **as well as in total menthol-sensitive neurons of menthol-sensitive/AIT-insensitive neurons (MS/AI, n = 23) and menthol-sensitive/AIT-sensitive (MS/AS, n = 7) neurons. **(E)**. Traces in **D **show menthol responsiveness (ΔF/F_0_) in MS/CI and MS/CS neurons. Traces in **E **show menthol responsiveness (ΔF/F_0_) in MS/AI and MS/AS neurons. ***F*) **Pie graph shows percentage distribution in total AIT-sensitive neurons of menthol-insensitive/AIT-sensitive neurons (MI/AS, n = 35) and menthol-sensitive/AIT-sensitive (MS/AS) neurons. Two traces show AIT responsiveness (Δ*F*/*F*_0_) in MI/AS and MS/AS neurons. Scale bar in each image is 20 μm. The horizontal bar in each trace indicates the 10-sec drug applications. Data are mean ± SEM in C, D, E.

Menthol-sensitive neurons were identified by calcium imaging following a brief application of 100 μM menthol for 10 seconds. These neurons consisted of 6.56% of total cells (n = 1475) in our DRG neuron cultures, a result consistent with our previous study [9]. We tested these menthol-sensitive neurons with capsaicin (0.5 μM, 10 sec) and AIT (100 μM, 10 sec) in order to see if, in some of them, TRPM8 receptors were also co-expressed with TRPV1 and TRPA1, two receptors believed to be expressed in nociceptive primary afferent neurons [12,27,28]. Based on their sensitivity to capsaicin and AIT, menthol-sensitive neurons could be classified into four subpopulations: **m**enthol-**s**ensitive/**c**apsaicin-**i**nsensitive (MS/CI, Figure 1A), **m**enthol-**s**ensitive/**c**apsaicin-**s**ensitive (MS/CS, Figure 1B), **m**enthol-**s**ensitive/**A**IT-**i**nsensitive (MS/AI, Figure 1A), and **m**enthol-**s**ensitive/**A**IT-**s**ensitive (MS/AS, Figure 1B). Of 71 menthol-sensitive neurons tested with capsaicin, 56% (40/71) were MS/CI neurons and 44% (31/71) were MS/CS neurons (Figure 1D). Of 30 menthol-sensitive neurons tested with AIT, 77% (23/30) were MS/AI and 23% (7/30) were MS/AS neurons (Figure 1E). Out of 7 neurons sensitive to menthol and AIT, 5 (71%) responded to capsaicin as well (Figure 1B) and thereby belonging to MS/CS neuron population [27,28]. In AIT-sensitive neurons, we also analyzed menthol-sensitivity to see if menthol sensitivity and AIT sensitivity were correlated in rat DRG neurons [29]). We found that the majority of AIT sensitive neurons (83%, 35/42) were insensitive to menthol and only small percentage of AIT-sensitive neurons (17%, 7/42) was menthol-sensitive (Figure 1F). Of 65 cells that responded to menthol and/or AIT, only 7 (11%) responded to both. The inverse correlation between menthol-sensitivity and AIT-sensitivity suggests that TRPA1 is unlike to significantly account for menthol-induced responses in our rat DRG neurons.

Menthol responses were analyzed and compared among subpopulations of menthol-sensitive neurons. Menthol responses were larger in MS/CI than in MS/CS (Figure 1D). Similarly, menthol responses were larger in MS/AI than in MS/AS (Figure 1E). The peak responses to 100 μM menthol, expressed as increases of Fluo-3 fluorescence intensity (Δ*F*/*Fo*), were 1.22 ± 0.13 (*n *= 40) for MS/CI and 0.70 ± 0.15 (*n *= 31, *P <*0.05) for MS/CS (Figure 1D); 1.11 ± 0.14 (*n *= 23) for MS/AI and 0.61 ± 0.14 (*n *= 7, *P *< 0.05) for MS/AS (Figure 1E). We also analyzed AIT responses in both MI/AS and MS/AS neurons. Kinetics of AIT responses in both groups was much slower (Figure 1F) than that of menthol-responses (Figure 1D,E). The average peak response (Δ*F*/*Fo*) to 100 μM AIT was 0.80 ± 0.72 (*n *= 35) for MI/AS neurons, significantly greater than that of that MS/AS neurons (0.43 ± 0.06, *n = *7) (Figure 1D, *P *< 0.05). Thus, AIT responsiveness also has inverse correlation with menthol-sensitivity.

**Figure 2:**
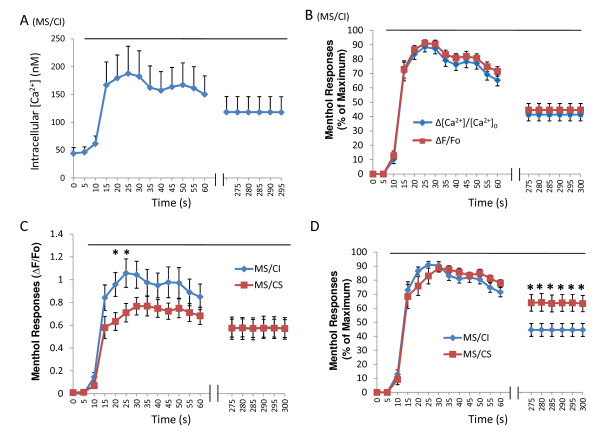
**Menthol responses in menthol-sensitive/capsaicin-insensitive and menthol-sensitive/capsaicin-sensitive neurons following prolonged menthol application**. **A**) Time course of the changes of intracellular Ca^2+ ^concentrations ([Ca^2+^]) in MS/CI neurons (n = 22) following the application of 100 μM menthol for 5 min. **B) **Menthol responses in MS/CI neurons expressed as percentages of maximal Δ[Ca^2+^]/[Ca^2+^]_0 _values or maximal ΔF/Fo values. **C) **Time course of menthol responses (ΔF/Fo) in MS/CI (n = 22) and MS/CS (n = 18) neurons during 5-min application of 100 μM menthol. **D**) Same as **C **except menthol responses at each time point are expressed as percent of peak menthol responses. In each experiment, menthol was continuously applied for 5 min. Capsaicin-sensitivity for each cell was tested 10 min after the termination of menthol applications. The horizontal bar in each figure indicates 5-min menthol application. Data are mean ± SEM; *P < 0.05.

Intrigued by the differences between MS/CI and MS/CS neurons in menthol responses to brief menthol applications, we examined whether there were also differences between these two subpopulations of neurons in their responses to prolonged menthol application (100 μM, 5 min). Changes in intracellular Ca^2+ ^concentrations ([Ca^2+^]) in MS/CI neurons following the prolonged application of menthol displayed adaptation, i.e., a gradual reduction of responses over time during prolonged menthol application (Figure 2A,B). In our study, values of Δ[Ca^2+^]/[Ca^2+^]_0 _were in a good agreement with the values of ΔF/Fo when menthol responses were expressed as percent of maximal responses (Figure 2B). Therefore, we used ΔF/Fo values as menthol responses for most of experiments. Photobleach was estimated to account for less than 1.2% reduction of fluorescent intensity at the end of 5-min menthol application (See additional file 1). During a 5-min menthol application, the peak response (ΔF/Fo) of MS/CI neurons (1.1 ± 0.10, n = 22 cells, 10 different dishes) was significantly larger than that of MS/CS neurons (0.71 ± 0.10, n = 18 cells, 10 different dishes, *P *< 0.05) (Figure 2C), a result similar to menthol response after brief menthol applications (Figure 1D). The prolonged menthol responses seems to be mainly due to Ca^2+ ^entry since menthol responses return near baseline when normal bath solution was replaced by a Ca^2+^- free bath solution during menthol application (see additional file 2). In both MS/CI and MS/CS neurons, menthol responses showed adaptation. Interestingly, in the last thirty seconds of 5-min menthol application, the response (Δ*F*/*Fo*) of MS/CI neurons was no longer larger than that of MS/CS neurons (Figure 2C), indicating a different degree of adaptation between MS/CI neurons and MS/CS neurons. The rate of adaptation could be more clearly observed when menthol response at each time point was expressed as percent of peak response (Figure 2D). For example, at the end of 5-min menthol application, the relative menthol response was 44.5 ± 4.6% (n = 22) in MS/CI neurons and was significant smaller than that in MS/CS neurons (63.8 ± 6.0%, *n = *18, P < 0.05). The differences between MS/CI and MS/CS neurons were unlikely due to their potential differences in buffering or extruding intracellular Ca^2+ ^because we did not observe a significant difference between the two groups of neurons in their responses to 5-min applications of 30 mM KCl (see additional file 3). Taken together, these results indicated that MS/CI neurons had a greater adaptation rate in menthol responses than MS/CS neurons (Figure 2D).

**Figure 3:**
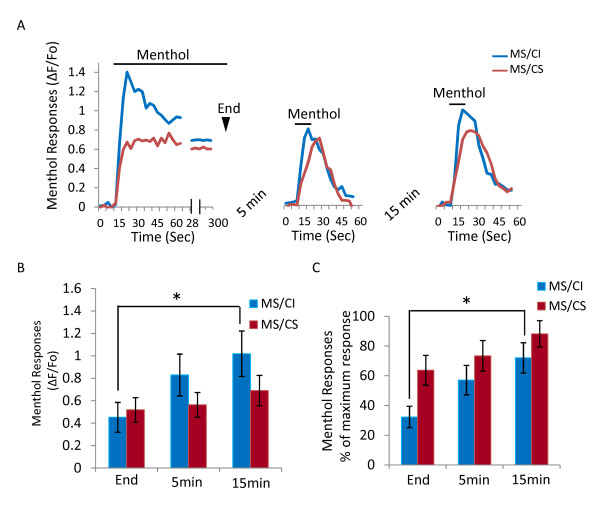
**Recovery after menthol-induced adaptation in menthol-sensitive/capsaicin-insensitive and menthol-sensitive/capsaicin-sensitive neurons**. **A) **Sample traces show menthol responses and adaptation in a MS/CI neuron and a MS/CS neuron following 5-min menthol application (left panel), recovery for 5 min (middle panel) and 15 min (right panel) in normal bath. The end of 5-min menthol application is indicated. Recovery was tested with short menthol application (10 sec). Menthol concentration was 100 μM for both prolonged and short applications. **B**) Pooled results show menthol responses (ΔF/F_0_) in MS/CI (n = 6) and MS/CS neurons (n = 8) at the end of 5-min menthol application, 5 min and 15 min recovery in normal bath. **C) **Similar to **B **except menthol responses were expressed as percent of maximum menthol responses observed during 5-min menthol application. Data are mean ± SEM in B and C; *P < 0.05.

**Figure 4:**
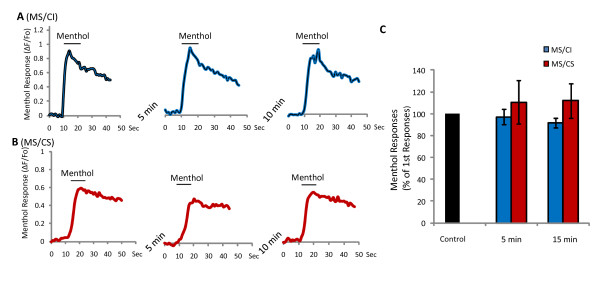
**Responses to multiple brief applications of menthol**. **A, B) **Sample traces of responses (ΔF/F_0_) to three brief applications of menthol (100 μM) in MS/CI (n = 12) and MS/CS (n = 11) neurons. Time interval was 5 min between first application and second application and was 10 min between second application and third application. Menthol was applied for 10 s each time. **C**) Summary of menthol responses. Data are mean ± SEM

Given the difference in peak response and adaptation rate between MS/CI and MS/CS neurons, we next examined recovery of menthol response after adaptation. This was achieved by testing menthol response to brief menthol application (100 μM, 10 s) at 5 and 15 minutes after the end of prolonged menthol application (Figure 3A). As shown in Figure 3B, C, menthol responses were 32.3 ± 7.2% (ΔF/Fo: 0.45 ± 0.13, n = 6 cells, 3 dishes) of peak responses in MS/CI at the end of 5-min menthol application. After washing cells in normal bath solution for 5 min, menthol responses slightly increased, but were not significantly different from the responses at the end of 5-min menthol application. After washing cells in normal bath solution for 15 min, menthol responses recovered to 72.0 ± 10.2% (ΔF/Fo: 1.02 ± 0.20, n = 6) of peak responses, significantly higher (p < 0.05) than the responses at the end of 5-min menthol application for MS/CI neurons. For MS/CS neurons, recovery from adaptation reached 88.2 ± 8.9% (n = 8 cells, 3 dishes) of peak responses after 15 min washing in normal bath solution. Brief menthol application (100 μM, 10 s) did not lead to any reduction of menthol response upon repeated menthol applications at 5 and 10 minute intervals in either MS/CI (*n = *12 cells, 7 dishes) or MS/CS neurons (*n = *11 cells, 7 dishes) (Figure 4). Thus, a time interval of longer than 5 min during multiple brief menthol applications is a suitable paradigm for testing recovery of menthol responses (Figure 3) and for some other experiments described below.

### Effects of protein kinase activators

**Figure 5:**
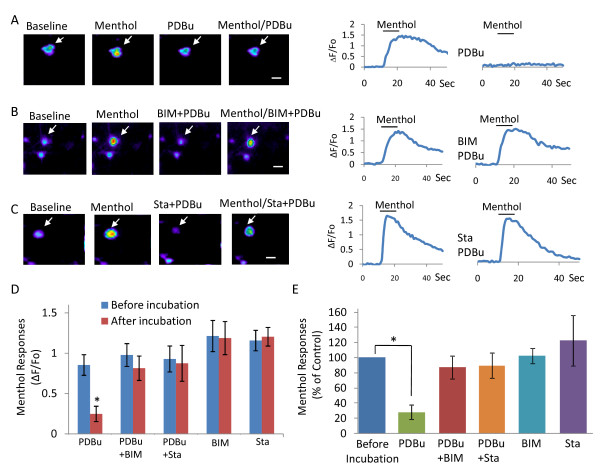
**Effect of PKC activator PDBu on menthol responses**. **A**) Sample images (left) and the trace to the right show fluorescence intensity in a cell at baseline, during menthol application, following treatment with 1 μM PDBu, and co-application of menthol with PDBu. **B) **Similar to **A **except BIM was applied together with PDBu and the test was on a different cell. **C**) Similar to **B **except staurosporine (Sta) was tested instead and the test was on a different cell. **D, E) **Pooled results show menthol responses before and after treatment with PDBu (n = 9), with PDBu in the presence of BIM (n = 13), with PDBu in the presence of Sta (n = 6), with BIM alone (n = 14), and with Sta alone (n = 24). Menthol responses were expressed as ΔF/F_0 _(**D**) and percent of menthol responses before treatment (**E**). In all cases 100 μM menthol was applied for 10 seconds. The PKC inhibitors BIM (1 μM) and Sta (0.5 μM) were pre-applied for 10 min and then co-applied with menthol. Maximum fluorescence intensity was used for menthol responses presented in **D **and **E**. Scale bars are 20 μm. Data are represented as mean ± SEM; *P < 0.05.

We examined whether in our MS/CI and MS/CS DRG neurons, direct activation of PKC with phorbol 12,13-dibutyrate (PDBu) significantly affect menthol-elicited responses. As shown in Figure 5, peak menthol response (ΔF/Fo) was reduced significantly from 0.86 ± 0.13 in control to 0.25 ± 0.10 (n = 9 cells, 3 dishes, *p *< 0.01) after treatment of cells with 1 μM PDBu for 5 min (Figure 5D). When expressed as percentage of peak response of control, menthol response was only 28.1 ± 9.8% of control following PDBu treatment (Figure 5E). The inhibitory effect of PDBu was observed in every cell tested regardless whether they were MS/CI (29.8 ± 16.1%, n = 5) or MS/CS neurons (26.0 ± 11.8%, n = 4). The effect of PDBu was most likely due to its activation of PKC since PDBu had no significant effect on menthol-elicited responses when cells were incubated, prior to the application of PDBu, with either the potent protein kinase inhibitor staurosporine (0.5 μM, 10 min) (ΔF/Fo: 0.88 ± 0.22, 89.79 ± 16.3% of control, *n = *6 cells, 2 dishes) or the specific PKC inhibitor BIM (1 μM, 10 min) (ΔF/Fo: 0.82 ± 0.15, 87.6 ± 14.9% of control, *n = *13 cells, 4 dishes) (Figure 5D,E). Menthol-induced responses were not significantly altered when cells were only treated with BIM (1 μM, 10 min) (ΔF/Fo: 1.19 ± 0.20, 102.5 ± 10.0% of control, *n = *14 cells, 4 dishes) or staurosporine (0.5 μM, 10 min) (ΔF/Fo: 1.22 ± 0.12, 123.0 ± 33.1% of control, *n = *24 cells, 7 dishes).

**Figure 6:**
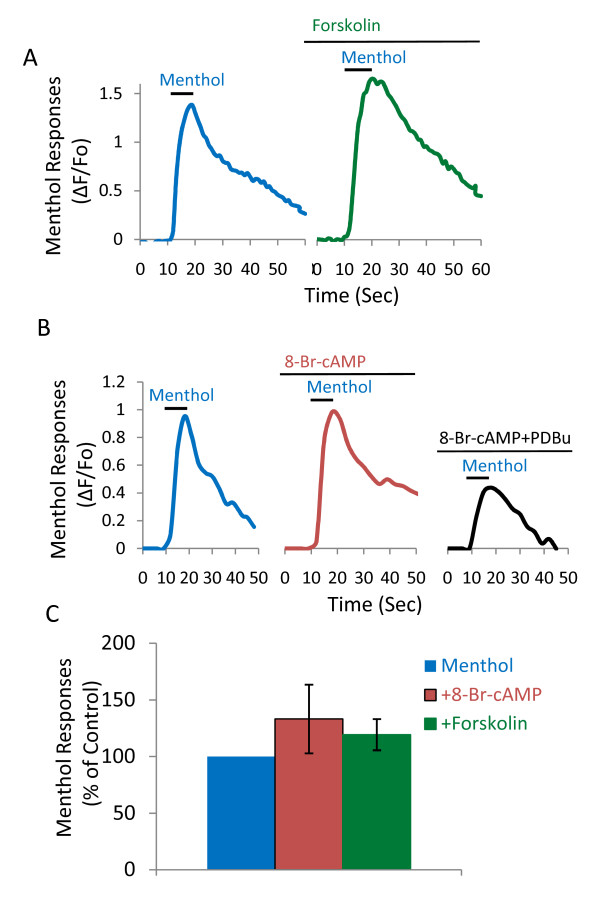
**Lack of effect by PKA activators on menthol responses**. **A, B) **Traces show menthol responses in a cell before and after treatment with 10 μM forskolin (10 min) (**A**) and in another cell before and after treatment with 100 μM 8-Br-cAMP (10 min) (**B**). Line breaks in the time axis are equivalent to 10 minutes. The cell in **B **was subsequently treated with 100 μM 8-Br-cAMP plus 1 μM μM PDBu. **C**) Summary of menthol responses before and after treatment with forskolin and 8-Br-cAMP. Menthol responses were expressed as percent of menthol responses before treatment (control). Menthol was applied for 10 seconds and peak fluorescence intensity was used. Data are represented as mean ± SEM.

We examined whether PKA activation may have an effect on menthol response (Figure 6). Menthol response was first tested by a brief menthol application (100 μM, 10 s) as control. Subsequently, PKA activator 8-Br-cAMP (100 μM) or Forskolin *(*10 μM) was applied to the cells for 10 min. Following this treatment, menthol response was tested again by a brief menthol application (100 μM, 10 s) in the presence of 8-Br-cAMP (100 μM) or Forskolin *(*10 μM). Menthol responses were 133.2 ± 30.3% (n = 8 cells, 3 dishes) and 119.3 ± 13.8% (n = 6 cells, 2 dishes) of controls following the treatment of cells with 8-Br-cAMP (100 μM) or forskolin respectively, which were not significantly different from control group (Figure 6C).

### Effects of protein kinase inhibitors

**Figure 7:**
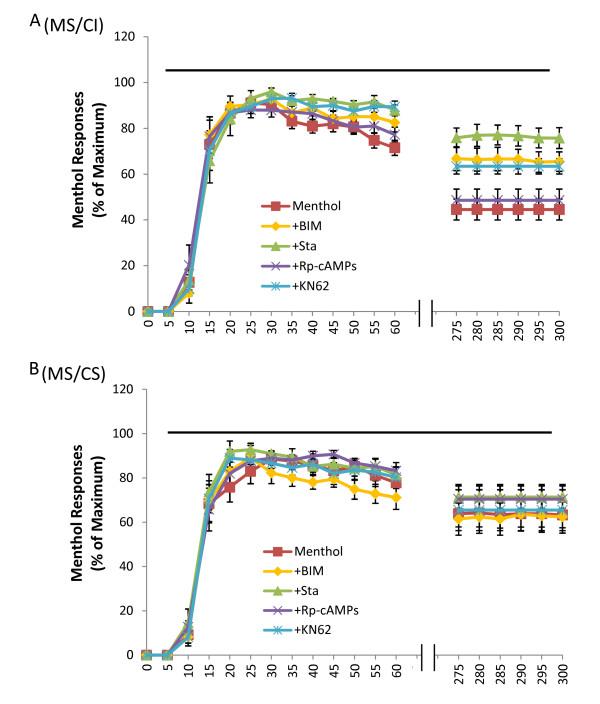
**Effect of protein kinase inhibitors on menthol responses in MS/CI and MS/CS neurons**. **A) **Responses of MS/CI neurons to prolonged menthol applications (100 μM, 5 min) in control group (red, n = 22) and groups treated with 1 μM BIM (orange, n = 10), 0.5 μM Sta. (green, n = 10), 25 μM KN62 (blue, n = 10), and 25 μM Rp-cAMP (purple, n = 14), P < 0.05. **B**) Responses of MS/CS neurons to prolonged menthol applications (100 μM, 5 min) in control group (red, n = 18) and groups treated with 1 μM BIM (orange, n = 10), 0.5 μM Sta. (green, n = 10), 25 μM KN62 (blue, n = 10), and 25 μM Rp-cAMP (purple, n = 10). In both A and B, menthol responses were expressed as percent of peak responses. The data for control groups in both A and B are taken from those in Figure 2D. The horizontal bar in each panel indicates duration of 5-min menthol application. Data are mean ± SEM.

We asked whether PKC, CaMKII, and PKA may play a role in shaping TRPM8-meidated responses during prolonged TRPM8 activation in both MS/CI and MS/CS neurons. This was achieved by testing effects of protein kinase inhibitors on menthol responses following prolonged menthol applications (Figure 7). In MS/CI neurons of control group for which cells were not treated with protein kinase inhibitors, menthol responses showed significant adaptation and the responses were reduced to 44.5 ± 4.6% (n = 22 cells, 10 dishes) of peak response at the end of 5-minute menthol application (Figure 7A, also Figure 2D). In the MS/CI neurons treated with staurosporine (0.5 μM, 10 min), PKC inhibitor BIM (1 μM, 10 min), or CaMKII inhibitor KN62 (25 μM, 10 minutes), the adaptation following prolonged menthol application was significantly attenuated (Figure 7A). For example, at the end of 5-minute menthol application, menthol-induced responses were 75.8 ± 4.3% (n = 10 cells, 5 dishes) when cells were treated with staurosporine, 66.7 ± 5.2% (n = 10 cells, 5 dishes) when cells were treated with BIM, 63.4 ± 4.9% (n = 10 cells, 3 dishes) when cells were treated with KN62; the responses under these conditions were all significantly larger than that of control group (P < 0.05). In the MS/CI neurons treated with PKA inhibitor RP-cAMPs (25 μM, 10 minutes), no significant difference was observed in menthol responses at any time points between control group and RP-cAMPs-treated group (Figure 7A). For example, menthol-evoked response was 48.6 ± 4.9% (n = 14 cells, 5 dishes) in RP-cAMPs-treated group at the end of 5-min menthol application, and was not significantly different from the control group (Figure 7A).

In MS/CS neurons, menthol responses were not significantly affected by the treatment of cells with any of the above protein kinase inhibitors (Figure 7B). For example, at the end of 5-minute menthol application, menthol responses were 61.4 ± 7.2% (*n = *10 cells, 4 dishes) in BIM-treated MS/CS group, 71.3 ± 5.0% (*n = *10 cells, 4 dishes) in staurosporine-treated MS/CS group, 65.4 ± 9.2% (*n = *10 cells, 3 dishes) in KN62-treated group, and 70.4 ± 6.6% (n = 10 cells, 5 dishes) in RP-cAMPs-treated group; the responses under above conditions were not significantly different from controls (63.8 ± 6.0%, *n = *18, 10 dishes) (Figure 7B).

## Discussion

In this study we demonstrated that menthol-sensitive/capsaicin-insensitive and menthol-sensitive/capsaicin-sensitive neurons had different degrees of responses and adaptation to menthol, that activation of protein kinase C, but not protein kinase A, resulted in a large reduction of menthol responses, and that protein kinase C and CamKII inhibitors had significant effects on menthol responses in MS/CI neurons but not in MS/CS neurons. These results reveal some new properties of TRPM8-mediated responses in both MS/CI and MS/CS neurons, the two neuron populations that most likely represent non-nociceptive cold-sensing neurons and nociceptive cold-sensing neurons respectively [9,30].

We used menthol as a TRPM8 agonist in the present study. Menthol has been found to interact with TRPA1 in heterologous expression systems that express mouse and human TRPA1 [29,31], raising a possibility that menthol responses in some cells may be mediated by TRPA1 in our study. However, using DRG neurons from rats, we found inverse rather than positive correlation between menthol-sensitivity and AIT-sensitivity (Figure 1E, F). Consistent with our results, low incidence of co-activation of menthol and AIT has been previously reported in DRG cells by others [28,32]. The low incidence of co-sensitivity to menthol and AIT is unlikely due to the failure of mentioned agonists to cross-activate these two receptors most of the times.

Therefore, TRPA1 is unlike to significantly account for menthol-induced responses in our rat DRG neurons. Menthol responsiveness was found to be higher in MS/CI neurons than in MS/CS neurons, a result consistent with our previous study using both the calcium imaging and patch-clamp recoding techniques on acutely dissociated DRG neurons [9,10]. Decay of menthol responses was found to be faster in MS/CI neurons than in MS/CS neurons, suggesting that MS/CI has faster adaptation and MS/CS has slower adaptation to menthol.

We showed that the PKC activator PDBu reduced menthol responsiveness in DRG neurons, and that this effect was abolished in the presence of staurosporine or BIM. Staurosporine inhibits a number of protein kinases including PKC, CaMKII, and tyrosine kinase (p60v-src), but BIM is a highly selective PKC inhibitor. The effect of these two inhibitors is consistent with a previous study that first suggested the involvement of PKC in regulating TRPM8 function in sensory neurons [18]. We further showed that menthol-induced adaptation could be significantly attenuated by staurosporine and BIM in MS/CI neurons. The inhibitor experiments added an important supplement to strengthen the argument that PKC plays a role in the adaptation of menthol responses in MS/CI neurons. In addition to PKC, we found that the selective CaMKII inhibitor KN62 attenuated adaptation of menthol responses in MS/CI neurons, suggesting that CaMKII may play a role in regulating TRPM8 functions in MS/CI neurons. In contrast to MS/CI neurons, menthol responses were not significantly affected by BIM, staurosporine, or KN62 in MS/CS neurons. We found that MS/CS neurons had smaller menthol response with weaker adaptation. It was initially thought that this property of MS/CS neurons might be a result of post-transcriptional regulation of TRPM8 function by protein kinases. However, the lack of the effects by protein kinase inhibitors on menthol responses in MS/CS neurons does not favor this idea. Alternatively, the smaller menthol responses in MS/CS neurons were due to the relatively lower TRPM8 expression as was proposed in our previous study [9]. The weaker adaptation observed in MS/CS neurons could be due to the smaller menthol responses in these neurons. However, we did not observe a clear co-relation between the degree of adaption and menthol responsiveness. Therefore, other factors might account for the differences in adaption rate between MS/CI and MS/CS neurons. One possible factor is PIP2, because PIP2 plays an important role in regulating TRPM8 functions and PIP2 hydrolysis accounts for menthol-induced desensitization [16,17]. It would be helpful in future studies to investigate whether PIP2 levels are significantly different between MS/CS and MS/CS neurons.

We explored potential involvement of PKA in regulating TRPM8 functions in rat DRG neurons by using both PKA activators and inhibitors. A previous study performed on HEK cells expressing TRPM8 showed that 8-Br-cAMP and forskolin, two PKA activators, inhibited TRPM8 activity induced by menthol [23]. However, both compounds were not found to have any significant effect on menthol responses in our work as well as in another recent study [22]. The discrepancy could be due to the use of different cell types. Direct inhibition of PKA by Rp-cAMP-S did not significantly affect menthol responses in our study, suggesting that there is no direct connection between PKA activity and TRPM8 functions. However, our result does not exclude the possibility that TRPM8 activity could be regulated indirectly through PKA pathway [22].

The differences in TRPM8-mediated responses and adaptation in nociceptive and non-nociceptive neuron populations may have physiological significances. Behavioral responses to innocuous and noxious cold stimuli are different and TRPM8-mediated adaptation may contribute to the differences. Mammals capably adapt to innocuous cold. On the other hand, noxious cold is poorly adapted, which perhaps is a conserved biological trait of mammalian sensory systems for animals to be aware of a harmful cold environment. TRPM8-mediated responses and adaptation in nociceptive and non-nociceptive neuron populations may only partially contribute to behavioral cold adaptation because other receptor molecules such as TRPA1 have been reported to also serve as cold sensors [27,33]. It would be interesting in future research to directly demonstrate differences to cold adaptation between nociceptive- and non-nociceptive cold sensing neurons and determine if such differences contribute to behavioral cold responses and adaptation.

## List of abbreviations

AIT: Allyl isothiocyanate; BIM: Bisindolylmaleimide; 8-Br-cAMP: 8-Bromoadenosine-3', 5'-cyclic monophosphate; CaMKII: Ca^2+^/calmodulin-dependent protein kinase; DMSO: Dimethyl sulfoxide; EGTA: Ethylene glycol bis(~-aminoethyl ether)-N,N,N',N'-tetraacetic acid; HBSS: Hank's Buffered Salt Solution; DRG: Dorsal root ganglion; KN-62: N,O-bis(5-isoquinolinesulfonyl)-N-methyl-L-tyrosyl]-4-phenylpiperazine; MS/CI: Menthol-sensitive capsaicin-insensitive; MS/CS: Menthol-sensitive capsaicin-sensitive; MS/AI: Menthol-sensitive AIT-insensitive; MS/AS: Menthol-sensitive AIT-sensitive; PDBu: Phorbol 12,13-dibutyrate; PIP2: phosphatidylinositol (4,5) bisphosphate; PLC: Phospholipase C; PKA: Protein kinase A; PKC: Protein kinase C; RP-cAMPs: Rp-Adenosine-3',5'-cyclic monophosphorothioate; S-MEM: Minimum essential medium for suspension culture; TG: Trigeminal ganglion; TRPM8: Transient receptor potential M8 (TRPM8) receptor; TRPV1: Transient receptor potential V1 receptor

## Competing interests

The authors declare that they have no competing interests.

## Authors' contributions

IS carried out the calcium imaging experiments, participated in the neuronal culture preparation and study design, performed the statistical analysis, and drafted the manuscript. JG conceived of the study, and participated in its design and coordination. All authors read and approved the final manuscript.

## Supplementary Material

Additional file 1 **Photobleach of DRG cells in calcium imaging experiments**. Changes in Fluo-3 intensity in MI/CI cells after 5-minutes Calcium imaging experiments. Click here for file

Additional file 2 **Menthol response reduction in Ca^2+^- free bath solution**. Menthol response (ΔF/Fo) in normal vs. Ca^2+^-free bath solution. Click here for file

Additional file 3 **DRGs response to KCL**. Calcium imaging of MS/CI and MS/CS neurons responding similarly to a 5-minute KCL application. Click here for file
