# Supplementary material for: Menthol response and adaptation in nociceptive-like and nonnociceptive-like neurons: role of protein kinases
Source: Mol Pain. 2010 Aug 20;6:47. doi: 10.1186/1744-8069-6-47 (PMC2936373; doi:10.1186/1744-8069-6-47)
Supplement: Additional file 1 — Photobleach of DRG cells in calcium imaging experiments. Changes in Fluo-3 intensity in MI/CI cells after 5-minutes Calcium imaging experiments. [file 1744-8069-6-47-S1.PPT]

## Slide 1
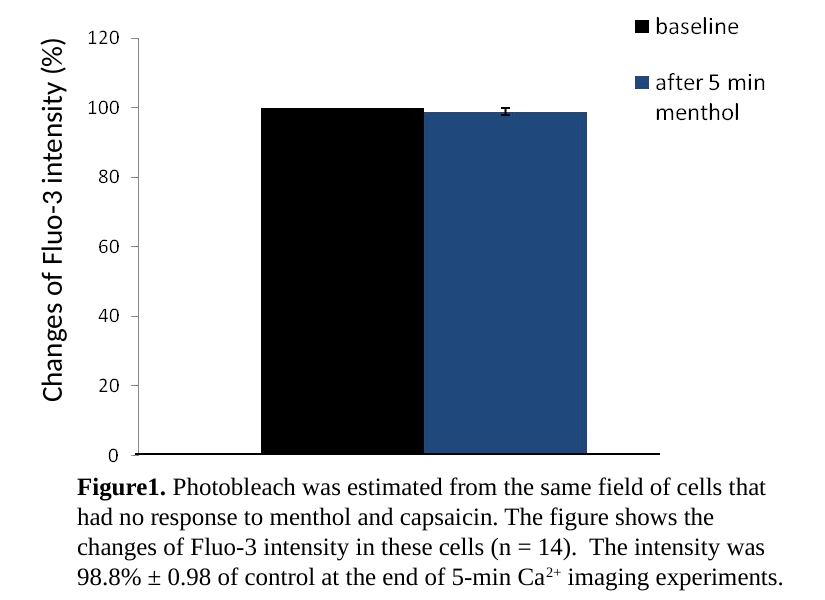

Changes of Fluo-3 intensity (%)
Figure1. Photobleach was estimated from the same field of cells that had no response to menthol and capsaicin. The figure shows the changes of Fluo-3 intensity in these cells (n = 14). The intensity was 98.8% ± 0.98 of control at the end of 5-min Ca2+ imaging experiments.
