# Supplementary material for: Menthol response and adaptation in nociceptive-like and nonnociceptive-like neurons: role of protein kinases
Source: Mol Pain. 2010 Aug 20;6:47. doi: 10.1186/1744-8069-6-47 (PMC2936373; doi:10.1186/1744-8069-6-47)
Supplement: Additional file 2 — Menthol response reduction in Ca2+- free bath solution. Menthol response (ΔF/Fo) in normal vs. Ca2+-free bath solution. [file 1744-8069-6-47-S2.PPT]

## Slide 1
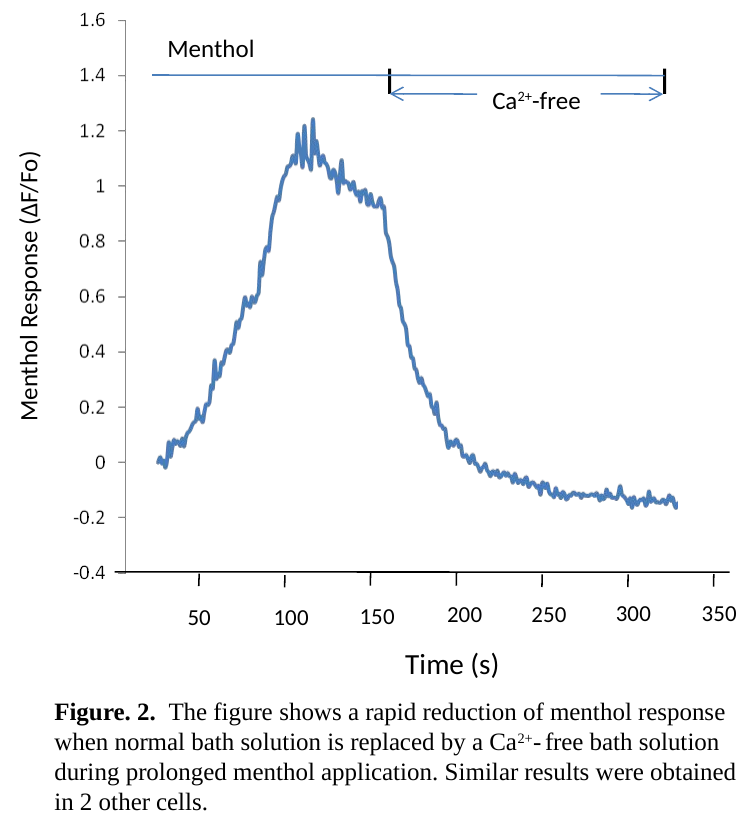

Menthol
Ca2+-free
Menthol Response (∆F/Fo)
300
350
200
250
150
50
100
Time (s)
Figure. 2. The figure shows a rapid reduction of menthol response when normal bath solution is replaced by a Ca2+- free bath solution during prolonged menthol application. Similar results were obtained in 2 other cells.
