# Supplementary material for: Menthol response and adaptation in nociceptive-like and nonnociceptive-like neurons: role of protein kinases
Source: Mol Pain. 2010 Aug 20;6:47. doi: 10.1186/1744-8069-6-47 (PMC2936373; doi:10.1186/1744-8069-6-47)
Supplement: Additional file 3 — DRGs response to KCL. Calcium imaging of MS/CI and MS/CS neurons responding similarly to a 5-minute KCL application. [file 1744-8069-6-47-S3.PPT]

## Slide 1
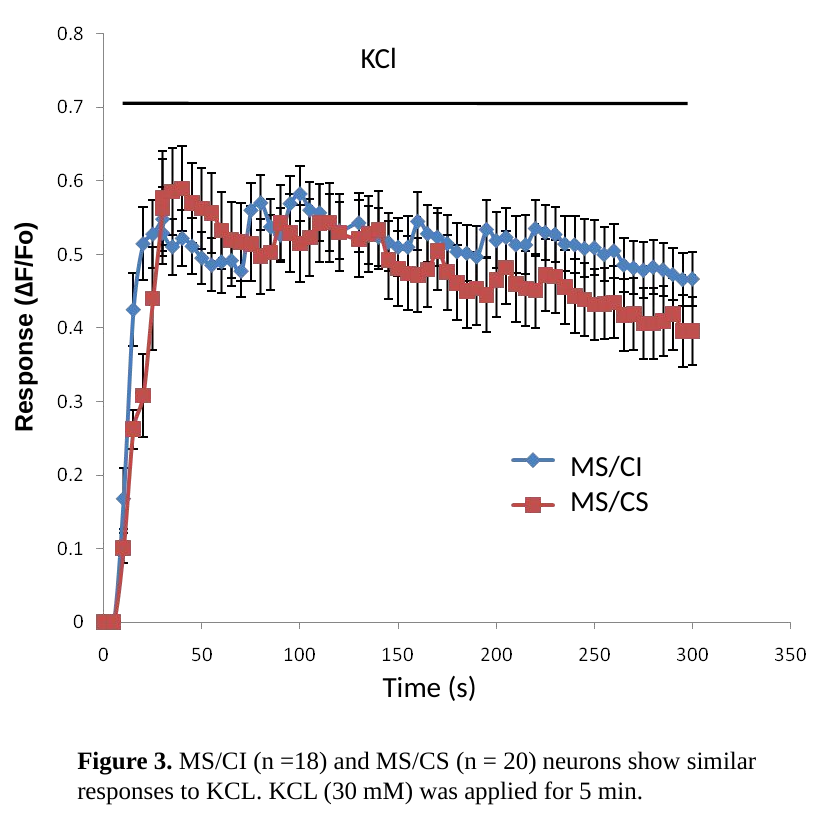

Response (∆F/Fo)
KCl
MS/CI
MS/CS
Time (s)
Figure 3. MS/CI (n =18) and MS/CS (n = 20) neurons show similar responses to KCL. KCL (30 mM) was applied for 5 min.
